# Impact of vitamin D supplementation on C-reactive protein; a systematic review and meta-analysis of randomized controlled trials

**DOI:** 10.1186/s40795-017-0207-6

**Published:** 2018-02-02

**Authors:** Mohsen Mazidi, Peyman Rezaie, Hassan Vatanparast

**Affiliations:** 1 Key State Laboratory of Molecular Developmental Biology, Institute of Genetics and Developmental Biology, Chinese Academy of Sciences, Chaoyang, Beijing China; 2 Institute of Genetics and Developmental Biology, International College, University of Chinese Academy of Science (IC-UCAS), West Beichen Road, Chaoyang, China; 3 Biochemistry and Nutrition Research Center, School of Medicine, Mashhad University of Medical Science, Mashhad, Iran; 4 College of Pharmacy and Nutrition, University of Saskatchewan, Health Sciences E-Wing, Clinic Place, Saskatoon, SK S7N 2Z4 Canada

**Keywords:** Meta-analysis, Vitamin D supplementation, C-reactive protein

## Abstract

**Background:**

To evaluate the effect of vitamin D supplementation on C-reactive protein (CRP) through a systematic review and meta-analysis of randomized control trials (RCTs).

**Methods:**

PubMed-Medline, SCOPUS, Google Scholar and Web of Science databases were searched (up until April 2016) to identify RCTs evaluating the impact of vitamin D supplementation on CRP. We used random effects models (using DerSimonian-Laird method) as well as the generic inverse variance methods for quantitative data synthesis. For sensitivity analysis, we applied leave-one-out approach. To examine the heterogeneity we used I2 index. Registration code: CRD42016036932.

**Results:**

Among 1274 search items**,** 24 studies met the inclusion criteria in the final evaluation. Pooling the data together indicated a non-significant decrease in CRP level following administration of vitamin D (weighted mean difference [WMD] -0.26(mg/l), (95% CI -0.75 to 0.22, *N* = 26 arms, heterogeneity *p* = 0.042; I^2^ 54.2%). The WMDs for IL6 was 0.67 pg/ml, (95% CI 0.29 to 1.06, *N* = 16 arms, heterogeneity *p* = 0.234; I^2^ 19.1%), 0.43 pg/ml, (95% CI 0.08 to 1.05, *N* = 26 arms, heterogeneity *p* = 0.120; I^2^ 42.1%), for IL10, and −0.11 pg/ml, (95% CI -0.53 to 0.30, *N* = 12 arms, heterogeneity *p* = 0.423; I^2^ 9.2%) for TNF-α, 4.03 pg/ml, (95% CI 3.50 to 4.57, *N* = 3 arms, heterogeneity *p* = 0.752; I^2^ 8.1%) for adiponectin. Sensitivity analyses confirmed the robustness of the findings.

**Conclusions:**

This study provided evidence that vitamin D supplementation had no impact on serum CRP, IL10, and TNF-α, while significantly increased serum IL6. We recommend RCTs with longer period of follow-up time (12 months) for future studies to provide explicit results.

**Electronic supplementary material:**

The online version of this article (10.1186/s40795-017-0207-6) contains supplementary material, which is available to authorized users.

## Background

Historically, vitamin D is recognised for its important role for bone health. However, recent studies suggest extra-skeletal effects of vitamin D through autocrine and paracrine systems. Low vitamin D concentrations are related with several diseases with inflammatory nature including rheumatoid arthritis, metabolic syndrome, type 2 diabetes, cardiovascular diseases, and some types of cancer [1]. Low vitamin D status is reported to to simulate mild acute phase response in which casue elevated concentrations of C-reactive protein (CRP), several hemostatic factors and different pro-inflammatory cytokines [2–4]. Studies suggest vitamin D suppelemnation may reduce circulating CRP levels and some other plasma inflammatory cytokines. However, inconsistent results are reported across completed randomized trials [5–7]. Cytokines such as interleukin 6 (IL-6), interleukin 10 (IL-10) and tumor necrosis factor- alpha (TNF-alpha) mediate the inflammatory response in human tehrfore they can serve as potential biomarkers of chronic inflammatory diseases [8–10]. The elevated circulating concentrations of pro-inflammatory cytokines, such as IL6, and hepatic acute phase proteins (e.g. CRP) is a common feature of such diseases with chronic inflammation [11, 12].

The potential effect of of vitamin D supplementation in decearsing chronic inflammation, if proven, is of public health interest given the disproportionate prevalence of vitamin D deficiency and insufficiency across the globe. A number of recent clinical trials have assessed vitamin D supplementation in different populations for its impact on circulating concentrations of several pro- and anti-inflammatory factors. However, such studies have had limitations such as small sample size, poor research design and subject traits (gender, ethnicity, age, etc.), and underpowered to achieve a comprehensive and reliable conclusion. Therfore there is substantial uncertainty about the net effect of vitamin D supplementation on CRP levels. Asystematic study which has addressed this issue dates back to 2014 inlcuding only a few studies [13]. Therefore, a comprehensive evalaution of evidence is needed to achieve an evidence-based conclusion. Hence, we aimed to address this uncertainty by systematically reviewing the literature, and meta-analysis of all trials, to explore the effects of vitamin D supplementation on CRP levels.

## Methods

We conducted his systematic review based on the international referred Reporting Items for Systematic Reviews and Meta- Analyses (PRISMA) Guidelines [13, 14]. We registed our study in the International Prospective Register of Systematic Reviews, PROSPERO (registration no: CRD42016036932).

### Literature search strategy

In litrature search we considred the effect of vitamin D supplementation on plasma CRP concentration as the primary exposure of interest. The secondary exposure was the effect of vitamin D supplementation on inflammatory and anti-inflammatory markers and cytokines. We considred multiple databases including PUBMED/ Medline, Cochrane Central Register of Controlled Trials (CCTR), Cochrane Database of Systematic Reviews (CDSR), Web of Science and Google Scholar, until April 2016 for litrature search. Weapplied relevant search terms to find a published and unpublished studies for our interested outcome (Additional file 1: Table S1). We conisdred no limitation on language.

### Selection criteria

We selected published randomized control trials (RCTs) assessing the impact of vitamin D administration on the infmlamatory parametres. Criteria for selecting studies: (i) clinical trial with single-arm, parallel or cross-over designs; (ii) RCTs of participants received vitamin D supplement compared to control group (either no vitamin D or placebo); and (iii) studies with information on primary outcome at the baseline and the endpoint in each group or the net change values. Exclusion criteria were: (i) non-clinical trials including those with case–control, cross-sectional or cohort designs; and (ii) studies missing to report mean (or median) plasma concentrations of our measures of interest at the baseline and/or at the endpoint. We carried out the selection by removing the of duplicates followed by titles and abstracts screening by two reviewers. The agreement between the reviewers was conisderable (Kappa index: 0.87; *p* < 0.001). We resolved the disagreements at a meeting between reviewers prior to selected articles being retrieved.

### Data extraction and management:

Two reviewers (MM, PR) retrieved the full text of studies the met the inclusion criteria, and screened to determine the eligibility. After assessment of methodological quality, the two reviewers extracted data onto a purpose-designed data extraction form. The same reviewers independently summarised the most significant results of each study f. We compared the summaries and any variations of ideas resolved through a discussion with the third reviewer (HV). Details information from selected RCTs is summarized in Table 1. An independent reviewer confirmed all data entries.

**Table 1:** General characteristics of the studies included

Author, year of publication	Country	Study design	Status	Sample size	Sex (% of women)	Mean age	Intervention	Supplemented the dose of vitamin D (IU/day)	Follow-up duration
A Sadiya (47), 2015	UAE	randomized double-blind clinical trial	vitamin D-deficient obese, type 2 diabetic	87	Male and Female (70%)	49 ± 8	cholecalciferol (vitamin D3)	phase 1; 6000 phase 2; 3000	6 month
A. Breslavsky (48), 2013	Israel	randomized, placebo-controlled	type 2 diabetes mellitus	47	Male and Female (53.1%)	66.8 ± 9.2	cholecalciferol (vitamin D3)	1000	12 month
Claudia Gagnon [34], 2014	Australia	randomized, placebo-controlled trial	vitamin D-deficient and at risk of type 2 diabetes	95	Male and Female (71%)	54 years	cholecalciferol (vitamin D3)	2000–6000	6 month
Edgar Turner Overton [33], 2015	USA	randomized, double-blind, placebo-controlled	HIV-infected	167	Male and Female (9%)	36 years	cholecalciferol (vitamin D3)	4000	48-week
Gavin Dreyer [30], 2014	UK	randomised controlled trial	non-diabetic chronic kidney disease stage 3–4 and concomitant vitamin D deficiency	38	Male and Female (39.1%)	45.8 (10.0)	ergocalciferol	50,000	6 month
Indrani Sinha-Hikim [25], 2015	USA	randomized	pre-diabetes and hypovitaminosis D	80	Male and Female (70%)	52.0 years	cholecalciferol (vitamin D3)	85,300 IU ± 16,000	12 month
Isa Gabriela de Medeiros Cavalcante [29], 2015	Brazil	double blind, randomized, placebo-controlled trial	With vitamin D insufficiency	40	Female (100%)	68 ± 6	cholecalciferol (vitamin D3)	200,000	4 week
Julia Åivo (49), 2015	Finland	double-blind, randomized, parallel		59	Male and Female (62.7%)	38 (22–53)	cholecalciferol (vitamin D3)	20,000	12 month
L. Wamberg (50), 2013	Denmark	double-blind design		52	Male and Female (71%)	18 to 50 years	cholecalciferol (vitamin D3)	7000	
M.D. Witham (51), 2015	UK	Parallel-group, double-blind, randomised placebo-controlled trial	with chronic fatigue syndrome	50	Male and Female (52%)	49 ± 13	cholecalciferol (vitamin D3)	100,000	6 month
M.P. BJORKMAN [28], 2009	Finland	randomised double-blind placebo controlled trial	chronically impaired mobility	218	Male and Female	84.5 ± 7.5	cholecalciferol (vitamin D3)	0 400 1200	6 month
Nafiseh Toghianifar (52), 2015	Iran	double blind randomized clinical trial	with a diagnosis of relapsing remitting multiple sclerosis (RRMS)	94	Male and Female (84.2%)	31.50 ± 7.60	cholecalciferol (vitamin D3)	50,000	12 week
Nasrin Sharifi (53), 2014	Iran	parallel, double-blind, placebo-controlled	non-alcoholic fatty liver disease (NAFLD)	53	Male and Female (51%)	40.33 ± 8.65	cholecalciferol (vitamin D3)	50,000	4 month
Ohk-Hyun Ryu [32], 2014	Korea	prospective, randomized, double-blinded, placebo-controlled trial	type 2 diabetic patients	62	Male and Female	54.5 ± 7.4	cholecalciferol (vitamin D3)	2000	24 week
Pamela R. von Hurst (54), 2010	New Zealand	randomised, placebo-controlled trial		81	Female (100%)	45.5	cholecalciferol (vitamin D3)	4000	6 month
Paulette D. Chandler (55), 2014	USA	Randomized, Placebo-Controlled Trial		328	Male and Female (67.7%)	51	cholecalciferol (vitamin D3)	1000 2000 4000	3 month
Rahaimi (56), 2013	Iran	randomised, placebo-controlled, double-blinded trial	With vitamin D deficiency	50	Female (100%)	30	cholecalciferol (vitamin D3)	50,000	2 month
Rolf Jorde (57), 2010	Norway	Randomized	overweight and obese	437	Male and Female (64.3%)	47	cholecalciferol (vitamin D3)	40,000	12 month
Seth I Sokol [31], 2012	USA	double-blind placebo wait-list control design	with CAD and vitamin D deficiency	90	Male and Female (26.5%)	55 ± 9.6	ergocalciferol	50,000	12 week
Tina K. Thethi, 2015	USA	double blind, randomized, placebo-controlled trial	with type 2 diabetes and chronic kidney disease	55	Male and Female (32.7%)	63	Paricalcitol	1 mcg	3 month
Tyler Barker (58), 2015	USA	randomized, double blind, placebo-controlled		56	Male and Female (32.7%)	32(7)	cholecalciferol (vitamin D3)	4000 8000	5 week
Ulla Kampmann (59), 2014	Denmark	double-blind, randomized, placebo-controlled trial	with type 2 diabetes and hypovitaminosis D	15	Male and Female (46.6%)	59.3 ± 4.4	cholecalciferol (vitamin D3)	5600 11,200	12 week
Zatollah Asemi (60), 2013	Iran	randomized, double-blind, placebo-controlled clinical	healthy pregnant women	48	Female (100%)	29	cholecalciferol (vitamin D3)	400	25 week

#### Quality assessment

We used the Cochrane criteria to assess potential bias [14].

#### Data preparation for meta-analysis:

According to Cochrane Handbook recommendations, the mean change from baseline in the level of variables of interest and standard deviation (SD) for both groups were collected and used to compute the effect size [15]. The following formula was used: SD = SEM × square root (*n*), where *n* is the number of subjects. We sued the GetData Graph Digitizer 2.24 [16] to extract the required data when theer were presented in graphs.

We applied random effects model (using the DerSimonian– Laird method) and the generic inverse variance method to take to account the heterogeneity of studies in terms of demographic characteristics of populations [17].A quantitaved assessment of Heterogeneity was conducted using I^**2**^ index, where values of 25%, 50%, and 75% reflect low, medium and high heterogeneity, respectively. We expressed the effect sizes as the weighted mean differences (WMD) and 95% confidence interval (CI). Sensetivity analysis was applied to assess the effct of each RCT on the overall effect size [18–20].

#### Determining potential publication bias

To determine potential publication bias we used Begg’s rank correlation, and Egger’s weighted regression tests /The fill’ and ‘fail-safe N’ and Duval & Tweedie ‘trim methods were applied to adjust for the potential effects of publication bias [21, 22]. The meta-analysis was preformed using Comprehensive Meta-Analysis (CMA) V3 software (Biostat, NJ) [23, 24].

## Results

### Selection RCTs

**Fig. 1:**
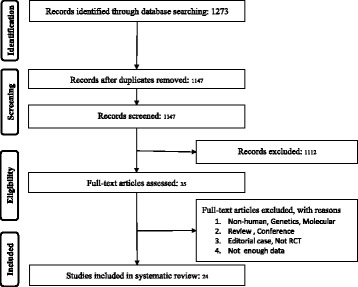
PRISMA flow chart for the studies selection

From searches in different search engines overall 1273 single citations recognized, of these, 126 were duplicates. From 1147 items, 35 left after assessment based on titles and abstracts, of which, 11 were not selected due to fact that: genetic, non-human studies, or molecular studies (*n* = 4); editorial or review articles (*n* = 3); incomplete data (2); Fig. 1. Consequently, 23 RCTs were used for pooling the data.

#### Risk of bias assessment

Results of assessment of bias revealed that some of the selected items recognized by the absence of information about the random sequence generation, blinding of outcome assessment and blinding of participants and study personnel, and allocation concealment. Though, nearly all of the assessed RCTs had a low risk of bias according to selective outcome reporting, with the exemption of two, which did not have sufficient material [25]. Details of the quality of bias assessment are presented in Additional file 1: Table S2.

### Characteristics of the studies

A summary of the characteristics of the studies is presented in Table 1. The inclused studies have been published between 2009 and 2015 from 12 countries including the United States of America (six studies), Iran (four studies), Finland, Denmark, UK (two studies) and Norway, Australia, Korea, UAE, New Zealand, Israel, Brazil (one study), respectively. The number of study participants ranged from 15 to 437 among studies.Four studies included only women; while the proportion of women in other studies ranged from 9% to 84.1%. The age of participants ranged from 18 to 92 years. The follow up duration from the baseline to endpoint across studies was from 4 weeks to one year. Various supplement regimens were assessed. Range of study population was from 15 [26] to 328 participants [27]. Twoenty one studies used cholecalciferol in a dosage range from 0 IU/d [28] to 200,000 IU/d [29]. In two of the studies, particciopants were summplemned with ergocalciferol at a dose of 50,000 IU at baseline for 26 [30] or 12 [31] weeks. In three studies, calcium supplements had also been administered in doses of 200 [32], 1000 [33], 1200 [34] mg/d, respectively.

#### Pooled estimate of the impact of vitamin D supplementation on C-reactive protein

**Fig. 2:**
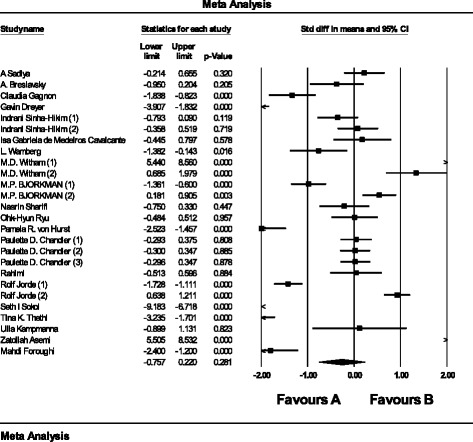
Weighted mean difference of the effect of vitamin D supplementation on C-reactive protein

The pooled estimate (weighted mean difference) of the effect of vitamin D supplementation on C-reactive protein was −0.26(mg/l), (95% CI -0.75 to 0.22, *N* = 26 arms, heterogeneity *p* = 0.042; I^2^ 54.2%) across all studies (Fig. 2). Further, we splited our data based on studies which followed their subjects >6 months and 6≤, respectively. This sub-analsyes changed the results as follows, −0.28(mg/l), (95% CI -0.44 to 0.12, I^2^ 22.1%) and −0.22(mg/l), (95% CI -0.33 to 0.11, I^2^ 20.9%) in more than six months and ≤6 months accordingly. We have divided our data based on mean age of the participants (>50 and 50≤), pooled estimate for >50 group was −0.75(mg/l), (95% CI -1.29 to −0.21, I^2^ 32.9%) and 50 ≤ −0.22(mg/l), (95% CI -0.36 to −0.07, I^2^ 25.9%). In terms of the sex, we ran the analysis for studies which included just females −0.34(mg/l), (95% CI -0.66 to 0.23, I^2^ 10.9%).

### Pooled estimate of the effect of vitamin D supplementation on IL-6

**Fig. 3:**
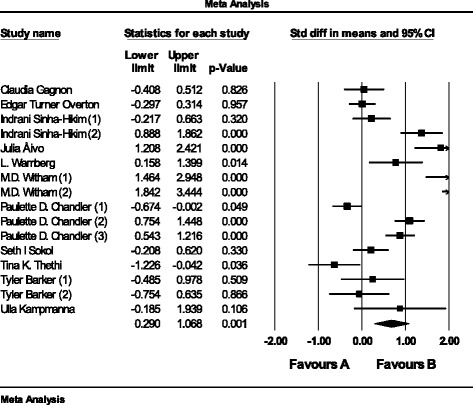
Weighted mean difference of the effect of vitamin D supplementation on IL-6

The pooled estimate (weighted mean difference) of the impact of vitamin D supplementation on IL-6 was 0.67 pg/ml, (95% CI 0.29 to 1.06, *n* = 16 arms, heterogeneity *p* = 0.234; I^2^ 19.1%) across all studies (Fig. 3).

#### Pooled estimate of the effect of vitamin D supplementation on IL-10

The pooled estimate (weighted mean difference) of the impact of vitamin D supplementation on IL-10 was 0.43 pg/ml, (95% CI -0.56 to 1.44, *N* = 9 arms, heterogeneity *p* = 0.120; I^2^ 42.1%) across all studies.

### Pooled estimate of the effect of vitamin D supplementation on TNF-α

The pooled estimate (weighted mean difference) of the impact of vitamin D supplementation on TNF-α was −0.11 pg/ml, (95% CI -0.53 to 0.30, *N* = 12 arms, heterogeneity *p* = 0.423; I^2^ 9.2%) across all studies.

### Pooled estimate of the effect of vitamin D supplementation on adiponectin

The pooled estimate (weighted mean difference) of the impact of vitamin D supplementation on Adiponectin was 4.03 pg/ml, (95% CI 3.50 to 4.57, *N* = 3 arms, heterogeneity *p* = 0.752; I^2^ 8.1%) across all studies.

### Pooled estimate of the effect of vitamin D supplementation on ICAM-1

The pooled estimate (weighted mean difference) of the impact of of vitamin D supplementation on ICAM-1 was −0.79 pg/ml, (95% CI 1.33 to 0.26, *N* = 4 arms, heterogeneity *p* < 0.001; I^2^ 62.1%) across all studies.

### Pooled estimate of the effect of vitamin D supplementation on IL-7

The pooled estimate (weighted mean difference) of the impact of vitamin D supplementation on IL-7 was −2.32 pg/ml, (95% CI -4.32 to −0.31, N = 4 arms, heterogeneity *p* = 0.635; I^2^ 7.9%) across all studies.

### Pooled estimate of the effect of vitamin D supplementation on IL-2

The pooled estimate (weighted mean difference) of the impact of vitamin D supplementation on IL-2 was −0.111 pg/ml, (95% CI -1.27 to 1.07, N = 4 arms, heterogeneity *p* = 0.826; I^2^ 6.3%) across all studies.

### Pooled estimate of the effect of vitamin D supplementation on IL-4

The pooled estimate (weighted mean difference) of theimpact of vitamin D supplementation on IL-4 was 0.027 pg/ml, (95% CI -0.72 to 0.77, *N* = 5 arms, heterogeneity *p* = 0.823; I^2^ 4.9%) across all studies.

### Pooled estimate of the effect of vitamin D supplementation on IL-5

The pooled estimate (weighted mean difference) of theimpact of vitamin D supplementation on IL-5 was 0.631 pg/ml, (95% CI -0.05 to 1.32, *N* = 5 arms, heterogeneity *p* = 0.425; I^2^ 5.8%) across all studies.

### Pooled estimate of the effect of vitamin D supplementation on IL-12

The pooled estimate (weighted mean difference) of theimpact of vitamin D supplementation on IL-12 was 0.045 pg/ml, (95% CI -0.14 to 0.23, N = 5 arms, heterogeneity *p* = 0.358; I^2^ 15.1%) across all studies.

### Pooled estimate of the effect of vitamin D supplementation on IL-13

The pooled estimate (weighted mean difference) of the impact of vitamin D supplementation on IL-13 was −0.15 pg/ml, (95% CI -0.78 to 0.48, N = 5 arms, heterogeneity *p* = 826; I^2^ 3.7%) across all studies.

### Sensitivity analysis

Tthe pooled effect estimates remained similar across all studies in leave-one-out sensitivity analyses (Table 2).

**Table 2:** Sensitivity analysis across all studies

Variables	Result of the leave-one-out sensitivity analyses
C-reactive protein	
Across all studies	-0.26(mg/l), (95% CI -0.75 to 0.22)
Interleukin-6	
Across all studies	0.67(ng/dl), (95% CI 0.29 to 1.06,)
Interleukin −10	
Across all studies	0.43(ng/dl), (95% CI -0.56 to 1.44)
TNF-α	
Across all studies	−0.11(ng/dl), (95% CI -0.53 to 0.30)
Adiponectin	
Across all studies	4.03 (pg/ml), (95% CI 3.50 to 4.57)
ICAM-1	
Across all studies	−0.79 (pg/ml), (95% CI 1.33 to 0.26)
IL-7	
Across all studies	−2.32 (pg/ml), (95% CI -4.32 to −0.31)
IL-2	
Across all studies	−0.111 (pg/ml), (95% CI -1.27 to 1.07)
IL-4	
Across all studies	0.027 (pg/ml), (95% CI -0.72 to 0.77)
IL-5	
Across all studies	0.631 (pg/ml), (95% CI -0.05 to 1.32)
IL-12	
Across all studies	0.045 (pg/ml), (95% CI -0.14 to 0.23)
IL-13	
Across all studies	−0.15 (pg/ml), (95% CI -0.78 to 0.48)
N=Number	

### Publication bias

**Fig. 4:**
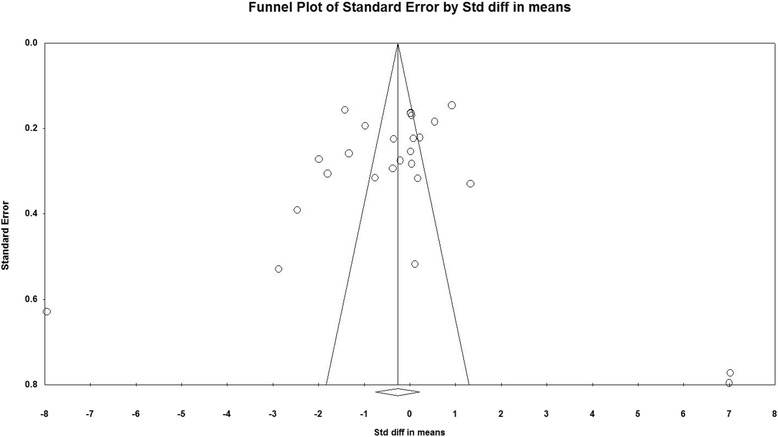
Funnel plot of standard error by Std difference in means

**Fig. 5:**
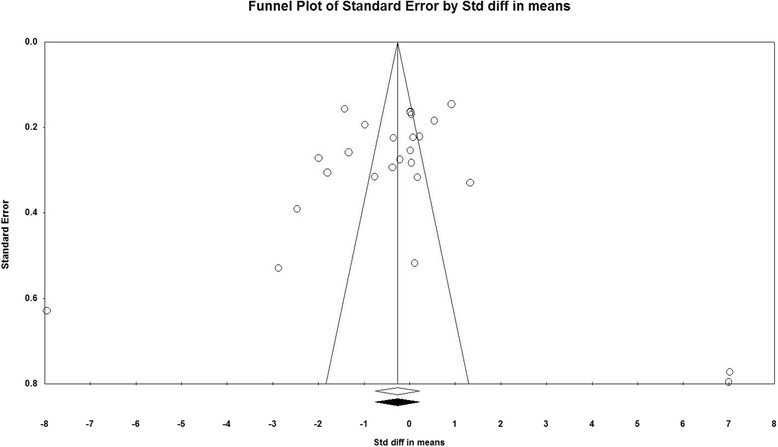
Funnel plot of standard error by Std difference in means

The visual inspection of funnel plot asymmetry declared no potential publication bias for the comparison of CRP levels between vitamin D supplementation and placebo groups (Fig. 4). Moreover, the presence of publication bias was not suggested by Egger’s linear regression (intercept = 2.12, standard error = 2.68; 95% CI = −3.41, 7.66, *t* = 0.79, df = 24.00, two-tailed *P* = 0.435) and Begg’s rank correlation test (Kendall’s Tau with continuity correction =0.04, z = 0.28, two-tailed *P* value =0.774). After adjustment of effect size for potential publication bias using the ‘trim and fill’ correction, no potentially missing studies were imputed in funnel plots. Hence no difference in effect size than the initial estimate (WMD −0.26(mg/l), 95% CI -0.75 to 0.22) (Fig. 5). The ‘fail-safe N’ test showed that 271 studies would be needed to bring the WMD down to a non-significant (*P* > 0.05) value.

## Discussion

In the current meta-analysis of randomized trials, we investigated the impact of high-dose vitamin D supplementation on circulating inflammatory and anti-inflammatory indexes. We detected no effect of vitamin D supplementation on circulating CRP. However our analysis revealed that vitamin D supplementation significantly increased IL-6 level by 0.67(pg/dl), while no significant effect was found on serum IL10 and TNF-α.

From a theoretical point of view, there are several possible mechanisms that may explain vitamin D may affect serum CRP and IL-6. The physiological impact of vitamin D is not limited to the homeostasis of calcium and phosphate. For instance, vitamin D receptors (VDR) play role in the decreased activation of the pro-inflammatory transcription factor nuclear factor kappa B (NF-κB). This suggests that VDR has an intrinsic inhibitory role in inflammation [35, 36]. One important target of vitamin D is NF-κB, which is inhibited by vitamin D, and via NF-κβ downstream release of the pro-inflammatory cytokines. NF-κB activation participates in the endogenous induction of CRP. Accordingly, the activated NF-κB may increase the effects of an activator of transcription-3 (STAT3) [37]. Studies have shown the active form of vitamin D (1,25-dihydroxyvitamin D3 [1,25(OH)2D] inhibits NF-κB activation. This inhibatory effect is done by upregulating the inhibitor of NF-κB (IκB-α) and reducing IkB-α phosphorylation in lipopolysaccharide-stimulated murine macrophage cells as well as submissively sensitized human airway smooth muscle cells [38, 39]. Thus, it may be hypothesised that vitamin D supplementation may suppress CRP via NF-κB and STAT3 signaling. Decreased parathyroid hormone (PTH) production with vitamin D supplementation may also explain the effects of vitamin D on hs-CRP. Low PTH may lead to decreased production of inflammatory factors [40].

According to our results, existing studies report mixed results regarding the impact of vitamin D supplementation on CRP. The Framingham Offspring Study cohort reported no significant association was found between vitamin D and CRP (*n* = 1381) [41]. While, in a meta-analysis of 10 randomized controlled trials (Chen et al. 2014), investigating the effect of vitamin D supplimentation on CRP [13], vitamin D supplementation significantly decreased the circulating CRP level by 1.08 mg/L [13]. In addition,, a recent meta-analysis of randomized controlled trials indicated a favourable impact on markers inflammation with vitamin D treatment [40]. It has been stated that heterogeneity across the findings of the studies may be due to supplemental dose of vitamin D, intervention durtaion and baseline hs-CRP level.

In the study by Forman et al.(2008) in a 1484 young women (aged 32 to 52 years)found that although vitamin D supplementation did not lower CRP specifically, it did lead to improvements in other inflammatory markers, for example IL-10 and TNF-α [42]. In addition, Ngo et al. studied 253 adults (aged 51 to 77 years) with mean CRP level of 3.6 ± 4.0 mg/mL and reported serum vitamin D have significant converse association with CRP level [43]. This association was seen in 147 morbidly obese subjects with CRP levels ranged from 1.88 to 4.01 mg/L [44]. In one study impact of vitamin D supplementation on CRP and IL-6 was different [45]. The one-year vitamin D supplementation in overweight and obese participants resulted in reduced serum IL-6 concentrations, while serum CRP concentrations were significantly increased. The contradictory findings in these studies may be attributed to the length of the study, seasonal change or geographical location [45]. In a randomized control trial in patients with acute myocardial infarction, a short duration of treatment with vitamin D has significant impact on weakening the rise of CRP and IL-6 (but not TNF-α) [46].

IL-6 is a multi-potential inflammatory cytokine that has a fundamental role in host defence including the immune responses, acute phase reactions and haematopoiesis [47]. Our analysis presented a positive association between vitamin D supplementation and circulating IL-6 levels. Our results may be influenced by seasonal differences in vitamin D level that cause changes in this increased levels of IL-6, IL-6-related signalling pathways, chronic diseases, congenital diseases, baseline IL-6 level, age, sex of subjects and a supplemental dose of vitamin D. Hence, this finding needs to be reexamined in larger randomized trials specifically designed to investigate the relationship between inflammatory indexes and vitamin D.

Our study has some potential limitations. Internal validity of our results relies on the quality of individual studies as it is seen in all meta-analsyes. Several limitations can be named in this regard. Firstly, most studies in this meta-analysis had medium sample sizes. This may lead to overestimation of vitamin D supplementaion effects. Smaller trials might be methodologically less robust and more prone to report larger effect sizes [48, 49]. The number of available studies on this topic was rather small. Only four of the studies inlcuded in current meta-analyses were with the duration of 12 months. Among them, only one had a relatively large sample. Heterogeneity exist in doses of vitamin and health status of target population at the baseline. Further, most of the studies were conducted in clinical population rather than general healthy population. This may likely affect the baseline levels of vitamin D and the inflammatory markers.

## Conclusion

The current study revealed that vitamin D supplementation significantly increase level of IL6, while having no effect on CRP, IL10, and TNF-α concentration. RCTs with larger sample size and longer follow-up period (12 months) should be considered for future investigations to provide an unequivocal answer.

## Additional file

Additional file 1: Table S1. Full search terms and strategy for the databases. **Table S2.** Quality of bias assessment of the included studies according to the Cochrane guidelines. (DOCX 26 kb)

## Abbreviations

CCTR: Cochrane Central Register of Controlled Trials
CDSR: Cochrane Database of Systematic Reviews
CDSR: randomized control trialsx
CI: confidence interval
CRP: C-reactive protein
IL-10: Interleukin 10
IL-6: Interleukin 6
NF-κB: Nuclear factor kappa B
PRISMA: Reporting Items for Systematic Reviews and Meta- Analyses
PROSPERO: International Prospective Register of Systematic Reviews
PTH: parathyroid hormone
SD: standard deviation
SEM: standard error of the mean
TNF-alpha: Tumour necrosis factor- alpha
VDR: Vitamin D receptors
WMD: weighted mean differences
