# Supplementary material for: Impact of vitamin D supplementation on C-reactive protein; a systematic review and meta-analysis of randomized controlled trials
Source: BMC Nutr. 2018 Feb 2;4:1. doi: 10.1186/s40795-017-0207-6 (PMC7050714; doi:10.1186/s40795-017-0207-6)
Supplement: Supplementary file 1 — Full search terms and strategy for the databases. Table S2. Quality of bias assessment of the included studies according to the Cochrane guidelines. (DOCX 26 kb) [file 40795_2017_207_MOESM1_ESM.docx]

**Supplementary Table1. full search terms and strategy for the databases**

| **No** | **Concept** | **Search terms** |
| --- | --- | --- |
| **1** | **Vitamin D** | Vitamin D[Text Word] OR vitamin d, 25-hydroxyvitamin D[Text Word] OR 25 hydroxyvitamin d[Text Word] OR cholecalciferol[Text Word] OR ergocalciferol[Text Word] OR 25-hydroxyvitamin D[Text Word] OR 25-hydroxy-vitamin D [Text Word] OR serum 25-hydroxyvitamin D[Text Word] OR serum 25-hydroxyvitamin D[Text Word] OR vitamin D2[Text Word] OR vitamin D3[Text Word] OR calcitriol[Text Word] OR calcidiol[Text Word]) OR calcifediol[Text Word] OR calciferol[Text Word] OR calciol[Text Word] OR calderol[Text Word] OR dihydrotachysterol[Text Word] OR dedrogyl[Text Word] OR dihydrotachysterol[Text Word] OR dihydroxycholecalciferol[Text Word] OR dihydroxyvitamin D[Text Word] OR dihydroxyvitamin D2[Text Word] OR dihydroxyvitamin D3[Text Word] OR doxercalciferol[Text Word] OR eldecalcitol[Text Word] OR ercalcidiol[Text Word] OR ergocalciferol*[Text Word] OR hidroferol[Text Word] OR hydroxycalciferol[Text Word] OR hydroxycolecalciferol[Text Word] OR hydroxycholecalciferol[Text Word] OR hydroxyergocalciferol*[Text Word] OR hydroxyvitamin D[Text Word] OR hydroxyvitamin D2[Text Word] OR hydroxyvitamin D3[Text Word] OR paricalcitol[Text Word] OR tachystin[Text Word] |
| **2** | **CRP** | ((((((high sensitivity C-reactive protein[MeSH Terms]) OR high-sensitivity C-reactive protein[MeSH Terms]) OR C-reactive protein[MeSH Terms]) OR high-sensitive C-reactive protein[MeSH Terms]) OR high sensitive C-reactive protein[MeSH Terms]) OR CRP[Title/Abstract]) OR hsCRP[Title/Abstract] |
| **3** | **Combination** | 1 AND 2 |

**Supplementary Table 2. Quality of bias assessment of the included studies according to the Cochrane guidelines.**

| Study | Random  sequence  generation | Allocation  concealment | Selective  reporting | Blinding of participants  and personnel | Blinding of outcome assessment | Incomplete  outcome data | Other  bias |
| --- | --- | --- | --- | --- | --- | --- | --- |
| **A Sadiya (**[**47**](#_ENREF_47)**), 2015** | U | U | L | U | U | L | L |
| **A. Breslavsky (**[**48**](#_ENREF_48)**), 2013** | U | H | L | L | U | L | L |
| **Claudia Gagnon (**[**31**](#_ENREF_31)**), 2014** |  |  |  | L | L |  |  |
| **Edgar Turner Overton (**[**30**](#_ENREF_30)**), 2015** | L | U | L | U | U | L | L |
| **Gavin Dreyer (**[**27**](#_ENREF_27)**), 2014** | L | L | L | L | L | L | L |
| **Indrani Sinha-Hikim (**[**22**](#_ENREF_22)**), 2015** | U | U | L | U | L | H | U |
| **Isa Gabriela de Medeiros Cavalcante (**[**26**](#_ENREF_26)**), 2015** | U | U | U | U | U | L | L |
| **Julia Åivo (**[**49**](#_ENREF_49)**), 2015** | U | U | L | U | U | L | L |
| **L. Wamberg (**[**50**](#_ENREF_50)**), 2013** | U | H | L | L | U | L | L |
| **M.D. Witham (**[**51**](#_ENREF_51)**), 2015** | U | L | L | L | L | H | L |
| **M.P. BJORKMAN (**[**25**](#_ENREF_25)**), 2009** | L | L | L | L | L | L | L |
| **Nafiseh Toghianifar (**[**52**](#_ENREF_52)**), 2015** | U | U | U | U | L | L | L |
| **Nasrin Sharifi (**[**53**](#_ENREF_53)**), 2014** | L | L | L | L | L | L | L |
| **Ohk-Hyun Ryu (**[**29**](#_ENREF_29)**), 2014** | H | L | L | U | U | L | L |
| **Pamela R. von Hurst (**[**54**](#_ENREF_54)**), 2010** | L | L | U | L | L | L | L |
| **Paulette D. Chandler (**[**55**](#_ENREF_55)**), 2014** | U | U | U | L | L | L | L |
| **Rahaimi (**[**56**](#_ENREF_56)**), 2013** | L | L | L | L | L | L | L |
| **Rolf Jorde (**[**57**](#_ENREF_57)**), 2010** | L | L | U | H | U | L | L |
| **Seth I Sokol (**[**28**](#_ENREF_28)**), 2012** | L | L | L | U | L | L | L |
| **Tina K. Thethi , 2015** | H | U | L | L | L | L | L |
| **Tyler Barker (**[**58**](#_ENREF_58)**), 2015** | L | L | L | L | U | L | L |
| **Ulla Kampmann (**[**59**](#_ENREF_59)**), 2014** | L | U | U | L | L | L | L |
| **Zatollah Asemi (**[**60**](#_ENREF_60)**), 2013** | U | L | L | U | L | L | L |

***L, low risk of bias; H, high risk of bias; U, unclear risk of bias.***
